# The effect of a palliative care pathway on medical interventions at the end of life: a pre-post-implementation study

**DOI:** 10.1007/s00520-022-07352-4

**Published:** 2022-09-07

**Authors:** Annemieke van der Padt-Pruijsten, Maria B. L. Leys, Esther Oomen-de Hoop, Agnes van der Heide, Carin C. D. van der Rijt

**Affiliations:** 1 Department of Internal Medicine, Maasstad Hospital, Rotterdam, the Netherlands; 2 Department Medical Oncology, Erasmus MC Cancer Institute, Rotterdam, the Netherlands; 3 Department of Public Health, Erasmus MC, University Medical Center Rotterdam, Rotterdam, the Netherlands

**Keywords:** Palliative care pathway, Cancer, Anticancer treatment, Medical interventions, End-of-life care

## Abstract

**Purpose:**

Adequate integration of palliative care in oncological care can improve the quality of life in patients with advanced cancer. Whether such integration affects the use of diagnostic procedures and medical interventions has not been studied extensively. We investigated the effect of the implementation of a standardized palliative care pathway in a hospital on the use of diagnostic procedures, anticancer treatment, and other medical interventions in patients with incurable cancer at the end of their life.

**Methods:**

In a pre- and post-intervention study, data were collected concerning adult patients with cancer who died between February 2014 and February 2015 (pre-PCP period) or between November 2015 and November 2016 (post-PCP period). We collected information on diagnostic procedures, anticancer treatments, and other medical interventions during the last 3 months of life.

**Results:**

We included 424 patients in the pre-PCP period and 426 in the post-PCP period. No differences in percentage of laboratory tests (85% vs 85%, *p* = 0.795) and radiological procedures (85% vs 82%, *p* = 0.246) were found between both groups. The percentage of patients who received anticancer treatment or other medical interventions was lower in the post-PCP period (40% vs 22%, *p* < 0.001; and 42% vs 29%, *p* < 0.001, respectively).

**Conclusions:**

Implementation of a PCP resulted in fewer medical interventions, including anticancer treatments, in the last 3 months of life. Implementation of the PCP may have created awareness among physicians of patients’ impending death, thereby supporting caregivers and patients to make appropriate decisions about medical treatment at the end of life.

**Trial registration number:**

Netherlands Trial Register; clinical trial number: NL 4400 (NTR4597); date registrated: 2014–04-27.

## Introduction

Diagnostic and therapeutical medical interventions can be used for seriously ill patients to prolong life and manage symptoms [1–3]. At the end of life, patients are frequently admitted to the hospital and often undergo multiple and costly medical interventions [4–7]. One can debate whether these interventions are always beneficial for patients with a limited life expectancy [6, 8]. In general, it is believed that aggressive care, e.g. the use of chemotherapy or admission to an intensive care unit, should be avoided at the end of life when possible [4–8]. Early integration of palliative care in oncological care has been suggested to improve quality of life in patients with advanced cancer [3, 9–12]. However, the effect of early integration of palliative care on the utilization of diagnostic procedures and medical interventions at the end of life has not been studied extensively.

In a cross-sectional study, end-of-life discussions about goals of care between healthcare professionals and patients have been found to be associated with less aggressive medical interventions at the end of life [7]. However, evaluation of the Serious Illness Care Program in patients with advanced cancer did not show a reduction in aggressive care or healthcare use at the end of life [8, 13, 14]. Many oncology healthcare professionals are not specialized in palliative care; however, they are responsible for the general coordination of palliative care in oncology patients. In the Netherlands, this role is formalized in the Dutch national quality framework of palliative care [15]. To support them to integrate palliative care more into their daily oncology practice, we developed a standardized palliative care pathway (PCP).

The PCP is a structured electronic medical checklist which supports healthcare professionals in integrating oncological and palliative care. We recently performed a pre-post intervention study on the effects of implementing this PCP in oncology departments in a large teaching hospital. Implementation of the PCP did not have a significant overall impact on place of death, hospitalizations at the end of life, and several aspects of advance care planning (ACP) [16]. However, in the group of patients for whom the PCP was actually used, more patients died outside the hospital compared to patients in the pre-PCP group [16]. These findings suggest that the PCP may have had an effect on decisions about clinical care.

The purpose of the present study was to investigate whether the PCP had an impact on medical care applied in patients with advanced cancer. We studied whether implementation of the PCP (1) affected the use of diagnostic procedures, anticancer treatment, and other medical interventions in patients’ last 3 months of life and (2) resulted in more involvement of a pain management team, a specialized palliative care team, and specialized psychosocial caregivers in patients’ last 3 months of life.

## Methods

### Design and study population

This study is part of a study performed in a large teaching hospital investigating the effects of implementing a standardized palliative care pathway (PCP) for patients with advanced cancer. In a pre- and post-intervention study, data were collected of adult patients with cancer who had been treated at the in- and outpatients clinics of the Departments of Oncology/Haematology and Lung Diseases and died between February 2014 and February 2015 (pre-PCP period) or between November 2015 and November 2016 (post-PCP period). Patients referred to other hospitals for further treatment were excluded.

During the 12-month pre-PCP period, care was provided as usual. At the end of this period, the PCP was implemented in departments involved. All nurses and physicians of the participating departments were trained on how to use the PCP in a 30–45-min training session; other hospital staff was informed in writing. We aimed to use the PCP for at least 50% of patients with cancer at the end of their life. To facilitate familiarity with the PCP, the post-PCP period started 9 months after implementation. The study design has been described elsewhere [16].

### Palliative care pathway

The PCP is a structured medical checklist based on Dutch and international guidelines for palliative care and ACP [17–19]. The pathway addresses all four domains of palliative care: physical, psychological, social, and spiritual. It is integrated in the patient’s electronic medical record, in which a special button guides the physician to the PCP. After opening, various prompts can be used, among these a prompt that offers guidance for healthcare professionals for ACP conversations and supports documentation of these conversations. Another prompt facilitates the coordination of care, e.g. for asking consultation of the pain management team, specialized palliative care team, and specialized psychosocial caregivers; the communication with the general practitioner; and involvement of family and relatives. The PCP can be used next to tumor-specific care pathways.

Indications to start the PCP were a negative answer to the surprise question [20] (‘would I be surprised if this patient would die within a year?’); deterioration of patient’s performance status; severe complication of a medical treatment; patient’s wish to stop all medical treatments; and/or no more anticancer treatment options available.

### Data collection

Data were collected retrospectively after patients’ death. Information was collected from their electronic medical records and included patients’ diagnosis, sociodemographic characteristics, and the use of diagnostic and medical interventions in their last 3 months of life (90 days). These included diagnostic procedures (laboratory tests such as blood sampling and urinalysis and radiology procedures); anticancer treatment (chemotherapy, radiotherapy, anti-hormonal therapy, immunotherapy, and surgery); other medical interventions (e.g. paracentesis, stenting, blood transfusions, and pleurodesis); and consultation of a pain management team, specialized palliative care team, and/or specialized psychosocial caregivers (spiritual counsellor, psychologist, social worker).

To promote consistency of data collection, 1 out of 20 electronic medical records were double checked by 2 different data collectors independently. Discrepancies were discussed and documented in a logbook. In case of a discrepancy, the particular outcome was adapted following the discussion and all medical records were checked for the parameter for which the discrepancy was found.

### Statistical analyses

Patients were included in either the pre- or post-PCP period; in the post-PCP period, patients were included irrespective of whether the PCP had been used. Furthermore, a per-protocol analysis was carried out, utilizing data from patients for whom the PCP was actually used during the post-PCP period. The statistical significance of differences in use of diagnostic procedures, medical interventions, and supportive care consultations between the pre- and post-PCP period was tested using *t*-tests, Mann–Whitney *U* tests, chi-square tests, or Fisher’s exact tests, where applicable. As the study concerned a secondary analysis of a larger study, a power analysis was not performed.

## Results

### Patients

We included 424 patients in the pre-PCP period and 426 patients in the post-PCP period; their mean age at death was 70.9 and 71.5 years, respectively. Both groups consisted of more males than females (58% and 56% were male). The most common primary cancer types were lung cancer, colorectal cancer, and haematological cancers (Table 1).

**Table 1:** Patient characteristics

	Pre-PCP (*N* = 424)	Post-PCP (*N* = 426)	*P*-value
	mean (SD)	mean (SD)	
Age at death	70.9 (11.2)	71.5 (10.8)	0.435
Gender	*** N* (%)**	*** N* (%)**	0.526
Male	248 (58)	240 (56)	
Female	176 (42)	186 (44)	
Primary cancer^a^			0.044
Lung	148 (34)	130 (29)	
Colorectal	56 (13)	50 (11)	
Haematological	39 (9)	68 (15)	
Gastric/oesophageal	38 (9)	39 (9)	
Breast	33 (8)	41 (9)	
Bile-pancreatic	30 (7)	38 (8)	
Prostate	31 (7)	30 (7)	
Urogenital (excl. prostate)	13 (3)	27 (6)	
Gynaecological	8 (2)	4 (1)	
Other	35 (8)	29 (6)	

^a^37 patients had 2 or 3 primary cancers

### Diagnostic procedures

In the last 90 days of life, most patients underwent multiple diagnostic procedures. Laboratory tests were performed in 85% of the patients in both periods with a median of 9.5 and 8 tests per patient in the pre- and post-PCP period, respectively. Further, 85% and 82% of the patients underwent radiology procedures, with a median number of 5 and 4 procedures per patient, respectively. Comparable results were found in the per-protocol analyses where (Table 2).

**Table 2:** Diagnostic procedures during patients’ last 90 days of life

	Pre-PCP (*N* = 424)	Post-PCP (*N* = 426)	*P*-value^b^	Post-PCP started (*N* = 236)	*P*-value^c^
Patients with laboratory tests (*n* (%))	362 (85)	361 (85)	0.795	208 (88)	0.322
Number of laboratory tests per patient (median-IQR^a^)	9.5 (4–19)	8 (4–16)	0.034	8 (4–16)	0.039
Patients with radiology procedures (*n* (%))	359 (85)	348 (82)	0.246	199 (84)	0.906
Number of radiology procedures per patient (median-IQR^a^)	5 (2–8)	4 (2–7)	0.130	4 (2–7)	0.123

^ a^*IQR*, interquartile range. ^b^*P*-value intention-to-treat analyses (pre-PCP period compared to the post-PCP period); Mann–Whitney U tests and chi-square tests were used. ^c^*P*-value per-protocol analyses (pre-PCP period compared to the post-PCP period only including patients for whom the PCP was started); Mann–Whitney U tests and chi-square tests were used

### Medical interventions

During the last 90 days of life, 40% of patients who died during the pre-PCP period received anticancer treatment, compared to 22% of the patients dying in the post-PCP period (*p* < 0.001). Additionally, significantly more patients in the pre-PCP period received systemic anticancer treatment in comparison to the patients in the post-PCP period (30% and 17%, respectively, *p* < 0.001), with chemotherapy as the main treatment used. In the pre-PCP group, more patients underwent local treatment, particularly radiotherapy (10% and 4%, respectively, *p* < 0.001). Comparable results were found in the per-protocol analyses (Table 3).

**Table 3:** Anticancer treatment during patients’ last 90 days of life

	Pre-PCP (*N* = 424)	Post-PCP (*N* = 426)	*P*-value^b^	Post-PCP started (*N* = 236)	*P*-value^c^
	*N (%)*	*N (%)*		*N (%)*	
Anticancer treatment	168 (40)	91 (22)	< 0.001	50 (21)	< 0.001
Systemic anticancer treatment^a^	129 (30)	74 (17)	< 0.001	42 (18)	< 0.001
Chemotherapy	102 (24)	61 (14)	< 0.001	34 (14)	0.003
Anti-hormonal therapy	19 (5)	9 (2)	0.057	6 (3)	0.288
Immunotherapy	12 (3)	9 (2)	0.517	6 (3)	1.000
Local anticancer treatment^a^	56 (13)	24 (6)	< 0.001	8 (3)	< 0.001
Radiotherapy	44 (10)	17 (4)	< 0.001	8 (3)	0.001
Surgery	12 (3)	7 (2)	0.257	0 (0)	0.006

^a ^Patients could receive multiple treatments; 6 patients received 2 types of treatment; the percentage for each anticancer treatment was calculated from the total population: pre-PCP *N* = 424 and post-PCP *N* = 426. ^b^*P*-value intention-to-treat analyses (pre-PCP period compared to the post-PCP period); chi-square test or Fisher’s exact test was used. ^c^*P*-value per-protocol analyses (pre-PCP period compared to the post-PCP period only including patients for whom the PCP was started); chi-square test or Fisher’s exact test was used

In the pre-PCP period, significantly more patients received medical interventions (other than anticancer treatment) (42%) compared with the post-PCP period (29%, *p* < 0.001). The two most frequently used medical interventions included paracentesis (16% and 13%, respectively) and blood transfusion (17% and 11%, respectively). In the pre-PCP period, patients more often underwent two or more interventions compared to the post-PCP period (26% and 14% respectively, *p* = 0.034). This difference was even more pronounced in the per-protocol analysis (26% and 7% respectively, *p* = 0.002) (Table 4).

**Table 4:** Other medical interventions during patients’ last 90 days of life

	Pre-PCP (*N* = 424)	Post-PCP (*N* = *426*)	*P*-value^c^	Post-PCP started (*N* = 236)	*P*-value^d^
	*N (%)*	*N (%)*		*N (%)*	
Medical interventions	180 (42)	125 (29)	< 0.001	67 (28)	< 0.001
Medical interventions (median-IQR)	1 (1–2)	1 (1–1)	0.081	1 (1–1)	0.006
Number of interventions per patient			0.034		0.002
1	127 (71)	100 (80)		59 (88)	
2	47 (26)	18 (14)		5 (7)	
3	6 (3)	6 (5)		2 (3)	
4	0 (0)	1 (1)		1 (1)	
Medical interventions — specific^a^			0.656		0.505
Paracentesis for ascites and thoracocentesis	69 (16)	55 (13)		30 (13)	
Blood transfusion	74 (17)	46 (11)		20 (8)	
Stenting (bile duct, oesophagus)	17 (4)	7 (2)		6 (3)	
Pleurodesis	14 (3)	8 (2)		2 (1)	
Other^b^	65 (15)	42 (10)		21 (8)	
Total	239	158		79	

^a ^Patients could undergo multiple treatments; the percentages of each specific medical intervention are calculated from the total population pre-PCP *N* = 424 and post-PCP *N* = 426. The *P*-value belongs to the comparison of these specific medical interventions between the pre- and post-PCP group. ^b^Includes e.g. treatment for symptom management: dyspnoea (morphine/oxygen) (N = 9); bisphosphonate infusions/denosumab (N = 6); dexamethasone (N = 7); antithrombotics (N = 4); recombinant human erythropoietin (N = 4) and operation for symptom management (N = 4, e.g. gamma nail pathological fracture); enteral tube feeding (N = 4); parenteral feeding (N = 3); percutaneous transhepatic biliary drainage (N = 3); nephrostomy tube (N = 3), (intention to) endoscopic intervention (N = 4), gastric tube (N = 2). ^c^*P*-value intention-to-treat analyses (pre-PCP period compared to the post-PCP period); chi-square test or Fisher’s exact test was used. ^d^*P*-value per-protocol analyses (pre-PCP period compared to the post-PCP period only including patients for whom the PCP was started); chi-square test or Fisher’s exact test was used

### Consultation of palliative care specialists and specialized psychosocial care

In the last 90 days of life, a pain management team was consulted in the pre- and post-period for 6% and 3% of the patients, respectively (*p* = 0.246), and a specialized palliative care team was consulted for 14% and 17%, respectively (*p* = 0.198). A spiritual counsellor was consulted for 23% of patients in the pre-PCP period compared to 19% in the post-PCP period (*p* = 0.141). In the group of patients for whom the PCP was started (*N* = 236), the palliative care team was consulted more often compared to the patient group in the pre-PCP period (14% vs 23%, respectively, *p* = 0.003) (Table 5).

**Table 5:** Consulted specialists and specialized psychosocial caregivers during patients’ last 90 days

	Pre-PCP (*N* = 424)	Post-PCP (*N* = 426)	*P*-value^a^	Post-PCP started (*N* = 236)	*P*-value^b^
	*N* (%)	*N* (%)		*N* (%)	
Pain management team	25 (6)	12 (3)	0.028	9 (4)	0.246
Palliative care team	60 (14)	74 (17)	0.198	55 (23)	0.003
Specialized psychosocial caregivers					
Spiritual counsellor	97 (23)	80 (19)	0.141	54 (23)	0.999
Psychosocial caregiver	33 (8)	26 (6)	0.335	18 (8)	1.000

^a^*P*-value intention-to-treat analyses (pre-PCP period compared to the post-PCP period); chi-square tests were used                                                                    ^b^ *P*-value per-protocol analyses (pre-PCP period compared to the post-PCP period only including patients for whom the PCP was started); chi-square tests were used

## Discussion

We found that significantly fewer patients received medical interventions in the last 3 months of life after implementation of a standardized PCP to support early integration of palliative care in general oncology care. The reduction was especially evident for the use of systemic and local anticancer therapies.

The percentage of patients receiving any kind of anticancer treatment in the last 3 months of life decreased from 40% during the pre-PCP period to 22% during the post-PCP period, which was mainly caused by fewer patients receiving chemotherapy (24% and 14%, respectively). Comparison of results with the results from other studies is difficult, as studies differ widely regarding the time frames studied, varying between 14 and 180 days before patients’ death [6, 21–24]. In a retrospective cohort study in cancer patients, the utilization of chemotherapy at least once in the last 180 to 30 days of life was analysed in seven developed countries, including the USA and the Netherlands. In the Netherlands, chemotherapy was used among 18.1% and 10.6% of patients, respectively, compared to 38.7% and 10.6% in the USA [25]. Nevertheless, the percentage of patients receiving chemotherapy in the post-PCP period in our study seems relatively low (14% in the last 90 days of life), compared to other studies, where 3 to 22.2% of patients were found to receive chemotherapy in the last 14 to 30 days before death [6, 21–24].

Our finding that fewer patients in the post-PCP period received local radiotherapy is noteworthy too. Radiotherapy with palliative intent may alleviate symptoms related to advanced cancer. Several studies have investigated radiotherapy use at the end of life and found rates between 6.4 and 28% in the last 30 days of life [26–28], which suggests that radiotherapy was relatively infrequently used in our study, both before and after implementation of the PCP (in 10 and 4%, respectively, in the last 3 months of life).

The utilization of other medical interventions, such as paracentesis (for ascites and thoracocentesis) and blood transfusion, was also lower in the post-PCP period (from overall 42 to 29%). In a retrospective cohort study, 10.1% of the patients with advanced cancer underwent paracentesis and 39.5% of the patients received blood transfusions in their last 30 days of life [27]. In our post-PCP group, only 13% of the patients underwent paracentesis and 11% of the patients received a blood transfusion in the last 90 days of their life.

In our study, patients underwent many diagnostic procedures in the last 3 months of life. Implementing the PCP was associated with a statistically significant reduction in the median number of laboratory tests per patient. No differences were found for the percentage of patients for whom laboratory tests or radiological procedures were performed. Comparing these results with other studies is difficult, because previous studies mainly evaluated the use of diagnostic procedures in the dying phase, i.e. the last days and hours of life [29–31]. Involvement of a pain management team, a specialized palliative care team, and specialized psychosocial caregivers did not increase after implementing the PCP. However, in the group in which the PCP was actually started, significantly more patients received care of a specialized palliative care team.

A previous paper on this study described that the implementation of the PCP did not result in a better documentation of ACP conversations, fewer hospitalizations at the end of life, or more out-of-hospital deaths [16]. The reduction of medical and, to some extent, diagnostic interventions that we found in the current study may nevertheless have been the result of increased awareness among healthcare staff of patients being in their last months of life. Such awareness may have been created by the extensive education of the healthcare professionals on using the PCP. Awareness of patients’ limited life expectancy has been found to result in fewer undesirable diagnostic procedures and medical interventions by others too [29–31]. End-of-life discussions and shifting to symptom-centred care goals have been associated with less utilization of anticancer treatment, including radiotherapy, in the last year of life [7, 32, 33]. The fact that we found comparable results in the intention-to-treat and the per-protocol analysis also suggests that implementation of the PCP created a general level of awareness about the importance of recognizing patients’ limited life expectancy.

Consultation of palliative care specialists and specialized psychosocial care may have contributed to the reduction of medical interventions. However, in the Netherlands, non-specialized healthcare professionals are responsible for the general coordination of palliative care, including the detection of specific palliative care needs for which palliative care specialists are consulted [15]. Given the rather large reduction in the use of anticancer treatment (from 40 to 22%), and the relatively small rise in consultations of palliative care specialists (from 14 to 17%), an increased awareness of healthcare staff seems more important [15].

This study is one of the few intervention studies in which healthcare professionals *not* specialized in palliative care were supported in giving structured palliative care and initiating ACP conversations. Previous similar studies mainly focused on the last weeks of life, whereas we measured the utilization of medical interventions in the last 3 months of life [13, 29]. There are several limitations to our study. Chemotherapy use in the last 14 days of life is suggested to be an indicator of ‘aggressive care’ [8, 13, 14, 21]. However, it is complex to distinguished appropriate versus inappropriate care at the end of life: interventions that can be considered ‘aggressive care’ for some patients can be used to manage and alleviate symptoms and suffering in others [1–3]. Prognostication of patients with advanced cancer is difficult on an individual basis and it is complex to predict which patients would benefit from a medical intervention [34]. Moreover, this study only collected data about medical interventions as provided in the hospital; information on interventions outside the hospital is lacking.

## Conclusion

In a prospective pre- and post-implementation study on a digital palliative care pathway (PCP) to support the integration of palliative care in oncology care, we found that implementation of the PCP resulted in fewer medical interventions, including anticancer treatments, in the hospital in the last 3 months of life.

Implementation of the PCP whether it was started or not may have created awareness among healthcare professionals of patients’ impending death and palliative care needs, and could support discussions about patients’ preferences and appropriate medical decision-making in the last phase of life. It is not possible to draw conclusions about the appropriateness or inappropriateness of decisions of medical interventions in our study. In future research, the appropriateness of medical intervention for patients with an advanced illness deserves more attention.

## Data Availability

The data of this study are kept by A. P. and are available upon request.
